# The essential effect of mTORC1-dependent lipophagy in non-alcoholic fatty liver disease

**DOI:** 10.3389/fphar.2023.1124003

**Published:** 2023-03-08

**Authors:** Xiangyun Tan, Xinyu Huang, Zhuhang Lu, Liang Chen, Junjie Hu, Xianxiang Tian, Zhenpeng Qiu

**Affiliations:** ^1^ College of Pharmacy, Hubei University of Chinese Medicine, Wuhan, China; ^2^ Hubei Key Laboratory of Resources and Chemistry of Chinese Medicine, Hubei University of Chinese Medicine, Wuhan, China

**Keywords:** NAFLD, lipophagy, mTORC1, hepatic steatosis, insulin resistance, hepatic fibrosis

## Abstract

Non-alcoholic fatty liver disease (NAFLD) is a chronic progressive liver disease with increasing prevalence. Lipophagy is a type of programmed cell death that plays an essential role in maintaining the body’s balance of fatty acid metabolism. However, the livers of NAFLD patients are abnormally dysregulated in lipophagy. mTORC1 is a critical negative regulator of lipophagy, which has been confirmed to participate in the process of lipophagy through various complex mechanisms. Therefore, targeting mTORC1 to restore failed autophagy may be an effective therapeutic strategy for NAFLD. This article reviews the main pathways through which mTORC1 participates in the formation of lipophagy and the intervention effect of mTORC1-regulated lipophagy in NAFLD, providing new therapeutic strategies for the prevention and treatment of NAFLD in the future.

## Introduction

Non-alcoholic fatty liver disease (NAFLD) caused by obesity and excessive/unbalanced diet has become a common liver disease worldwide. According to statistical and epidemiological analysis, 1.9 billion people worldwide suffer from NAFLD, accounting for about 25% of the world’s population (Younossi et al., 2018; Younossi et al., 2019). The etiology of NAFLD is complex and accompanied by multiple complications. Therefore, the development of suitable therapies is slow, and the number of candidate drugs that have successfully entered the late clinical stage is small and challenging to develop. A healthy lifestyle remains the key to prevention and treatment (Romero-Gomez et al., 2017; Sheka et al., 2020). There is an urgency to explore practical strategies for treating NAFLD.

NAFLD is a metabolic stress-induced liver injury disease, mainly manifested by steatosis and lipid accumulation in hepatic parenchymal cells. According to the course of the disease, it is divided into simple fatty liver, steatohepatitis, liver fibrosis, and hepatocellular carcinoma (Marengo et al., 2016; Nassir, 2022). The link between the main disease features of NAFLD and the actual events leading to hepatic cell death—insulin resistance, lipid metabolism imbalance, inflammation, and oxidative stress—is increasingly recognized (Zhao et al., 2021). Programmed cell death is a gene-directed form of cell self-extinction, which is a necessary condition for the normal development of biological tissues, including apoptosis, autophagy, pyroptosis, necroptosis, ferroptosis, and cuproptosis (Lockshin, 2016; Bedoui et al., 2020; Kopeina and Zhivotovsky, 2022). Multiple studies have found that lipophagy, a form of selective autophagy, is significantly inhibited during the development of NAFLD (Chen and Lin, 2022). Alleviating lipid metabolism disorders by restoring lipophagy dysregulation is an effective strategy to prevent and treat NAFLD. Furthermore, the mammalian target of rapamycin (mTOR), an atypical serine/threonine protein kinase and a member of the phosphatidylinositol kinase-related kinase (PIKK) family, plays an important role in NAFLD as Table 1 shows. mTOR exists in two complexes, the target of rapamycin complex 1 (mTORC1) and rapamycin complex 2 (mTORC2). Among them, mTORC1 is a valuable negative core lipophagy regulator (Kim and Guan, 2015; Liu et al., 2022).

**TABLE 1:** The effect of and the research development of mTOR in NAFLD.

mTOR-associated signaling pathway	Compound or Intervention	Main finding in NAFLD	Reference
mTOR/S6K1	lncRNA-NEAT1	Reduce lipid accumulation in the liver	Wang (2018)
mTORC1/S6K/SREBP1	PPDPF-LKO	Inhibit mTOR signaling activation, reduce hepatic steatosis	Ma et al. (2021)
DEPTOR/mTOR	Neddylation inhibition	Promote fatty acid oxidation, improve lipid denaturation	Serrano-Macia et al. (2021)
p-AKT/mTOR/LC-3II	Probiotics and Metformin	Targeting intestinal ecological dysregulation, inflammatory and the autophagy pathway to prevent NAFLD progression and liver injury	Seif El-Din et al. (2021)
Nrf2/AMPK/mTOR	Pterostilbene	Alleviate oxidative stress caused by excessive lipid accumulation, promote the metabolism and decomposition of fatty acids	Shen et al. (2023)
	Geniposide	Reduce lipid accumulation, enhance resistance to oxidative stress and anti-inflammatory capacity	Shen et al. (2020)
AMPK/mTOR	Empagliflozin	Improve liver damage and disorders of lipid metabolism	Meng et al. (2021)
	Kangtaizhi Granule	Improve lipid accumulation and hepatic steatosis	Zhang et al. (2020)
	Dapagliflozin	Activate autophagy, improve liver steatosis	Li et al. (2021); Sun et al. (2015); Porcu et al. (2018)
	Hydrogen sulfide		
	Oleuropein		
	Schisandrin B	Reduce hepatic steatosis and promote fatty acid oxidation	Yan et al. (2022); Lee et al. (2020)
	Citrus Peel		
AKT/mTOR	Pueraria flavonoids	Induce autophagy, reduce lipid accumulation and inflammation levels	Sun et al. (2023)
	Follistatin	Relieve hepatic steatosis, reduce DNL	Tong et al. (2022)
	Caveolin-1	Reduce lipid accumulation, promote autophagy	Xue et al. (2020)
AKT/mTOR/SREBP-1	Ursodeoxycholic acid	Inhibit hepatic steatosis	Hu et al. (2019)
mTORC1/SREBP-1c	Oleuropein	Improve lipid accumulation and insulin resistance	Porcu et al. (2018)
AMPK/mTOR/SREBP-1	Betulinic acid	Improve lipid accumulation in liver cells	Quan et al. (2013)
LKB12/AMPK/mTOR	γ-Linolenic Acid	Regulate lipid metabolism and autophagy	Liang et al. (2021)
mTORC1	Korean red ginseng	Inhibit steatosis, liver damage and inflammation	Choi et al. (2019)
	Acetyl-CoA	Activate autophagy	He et al. (2020)

This article briefly discusses the role of mTORC1 in the formation of lipophagy, summarizes its intervention effect on the regulation of lipophagy in NAFLD, and preliminarily clarifies the relationship between mTORC1 and lipophagy and NAFLD.

## Overview of lipophagy

The term “autophagy” was first introduced by Christian de Duve at the International Conference on Lysosomes in 1963 (Klionsky, 2008; Wen and Klionsky, 2020). More than 20 years later, Yoshinori Ohsumi gradually explored the physiological significance and molecular mechanism of autophagy, laying a solid foundation for the field of autophagy (Tsukada and Ohsumi, 1993; Ohsumi, 2014).

Autophagy is a phenomenon of cellular degradation within lysosomes of defective or aged organelles and proteins, invading pathogens and some cytoplasmic components after receiving induction signals from autophagy-related genes, and is also considered type II programmed cell death (Levine and Klionsky, 2004; Li et al., 2020b). There are three known pathways of cellular autophagy: macroautophagy, microautophagy, and molecular chaperone-mediated autophagy. In general, substrates are classified according to the size and type of the degraded substrate, the scale of their degradation, and the various mechanisms by which the substrate is transported to the lysosome (Parzych and Klionsky, 2014; Bednarczyk et al., 2018; Mizushima and Levine, 2020). Macroautophagy is present in all eukaryotes and is the dominant form of autophagy. Unless otherwise stated, the term “autophagy” generally refers to macroautophagy (Yang and Klionsky, 2009).

Lipophagy is the process in which cells selectively recognize and degrade cholesteryl esters and triglycerides in lipid droplets (LDs) by activating autophagy-associated molecules to form free cholesterol, fatty acids, and glycerol to prevent excessive accumulation of lipids (Schulze et al., 2017). The co-localization of autophagy markers and LDs markers in hepatocytes and the involvement of autophagy in the clearance of lipid droplets and triglycerides identified for the first record the presence of lipophagy (Singh et al., 2009). The series of processes involved in lipophagy is that under the stimulation of internal and external factors, a membrane vesicle with a double-layer membrane structure appears in the cytoplasm, which gradually bends into a cup-like vesicle system through continuous extension and expansion: autophagic precursors. The autophagic precursor can enclose the nearby LDs and form an autophagosome after complete closure. The outer membrane of the autophagosome fuses with the lysosome and enters the lysosome with a monolayer structure. Inside the lysosome, the monolayer structure and contents of the autophagosome can be rapidly degraded by the action of related enzymes and eventually degraded to free fatty acids and glycerol. (Kim et al., 2001; Shin, 2020; Li and Peng, 2022).

## Molecular mechanisms of mTORC1 involved in the formation of lipophagy

**FIGURE 1:**
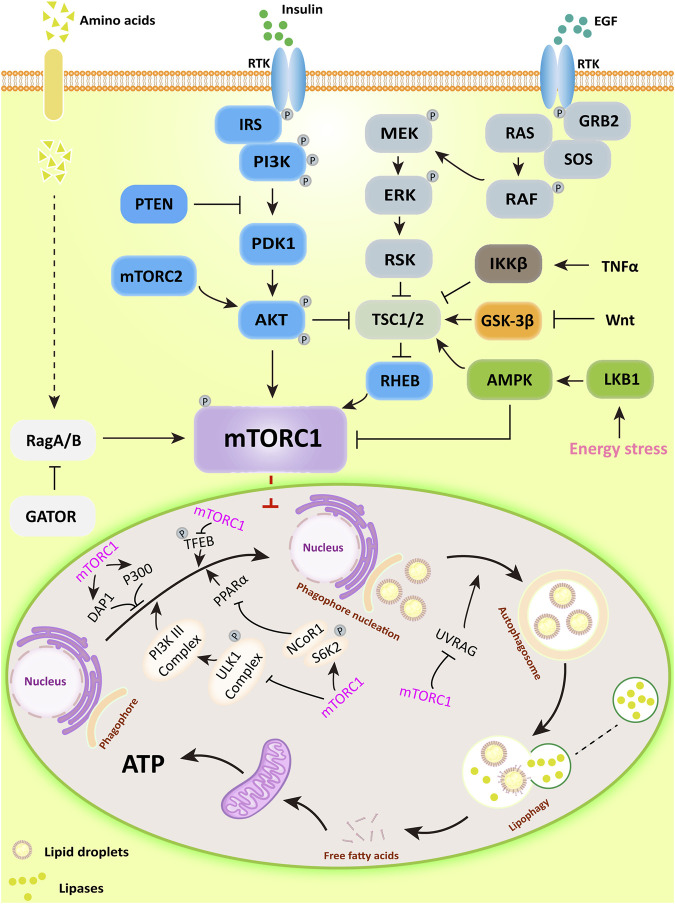
The major upstream regulatory pathways of mTORC1 and its main mechanisms for regulating lipophagy. PI3K/AKT activates downstream mTORC1 by directly phosphorylating or inhibiting TSC1/2, a potent mTOR inhibitor; AMPK can directly inhibit mTORC1 activity or inactivate mTORC1 in a TSC1/2-dependent manner. Subsequently, mTORC1 regulates lipophagic processes by regulating the ULK1 complex, DAP1, and P300-mediated acetylation.

mTORC1 is a negative regulator of lipophagy, the main upstream regulatory pathways (PI3K/AKT, RAS/RAF, AMPK, Wnt, and TNF) and its function in lipophagy are shown in Figure 1.

mTORC1 inhibits lipophagy by affecting autophagosome formation. The ATG kinase complex induces autophagosome formation in yeast macrophages (Kamada et al., 2000). In mammalian cells, the complex consists of the Unc-51 serine/threonine kinase family ULK1/ULK2, ATG13, and 200 kDa adhesion point kinase family interaction proteins (FIP200) (Hara et al., 2008; Ganley et al., 2009; Kim et al., 2011). ULK1/2 is a downstream target of mTORC1, their binding interaction is influenced by nutritional status (Hosokawa et al., 2009b; Nagy et al., 2014). Under nutrient-rich conditions, mTORC1 binds to the complex and dissociates from nutrient-deficient states. When mTORC1 associates with the complex, it can inactivate ULK1/2 and ATG13 by inducing their phosphorylation. When cells are treated with the mTOR inhibitor rapamycin or are undernourished, mTORC1 dissociates from the ULK complex, stimulating the process of lipophagy (Hosokawa et al., 2009a; Jung et al., 2009).

In addition, the Class Ⅲ PI3K complex, a downstream regulator of the ULK1 complex, is also significant for the formation of autophagosomes (Longatti and Tooze, 2009). This complex consists of PIK3C3/VPS34, PIK3R4/p150, and Beclin-1 in mammalian cells (Kihara et al., 2001; Furuya et al., 2005). Beclin-1 binds to Class III PI3K to form the Beclin-1-PIK3C3/VPS34 complex, the effect of which depends mainly on the status and activity of Beclin-1 (Kang et al., 2011). For example, the anti-apoptotic protein Bcl-2 binds Beclin −1 and prevents its interaction with PIK3C3, and the Beclin −1 binding protein Rubicon inhibits PIK3C3 activity in Class III PI3K complex (Pattingre et al., 2005; Matsunaga et al., 2009). In addition, SH3GLB1/Bif-1, a positive regulator of the Class III PI3K complex, promotes autophagy by facilitating the interaction of UVRAG with Beclin-1 (Takahashi et al., 2007). AMBRA1, another positive regulator of the Class III PI3K complex, also promotes autophagosome nucleation directly by facilitating the exchange of Beclin-1 with the kinase VPS34 (Di Bartolomeo et al., 2010). Moreover, mTORC1 can directly phosphorylate AMBRA1 to inhibit lipophagy and also directly phosphorylate ATG14 in the Class III PI3K complex through several sites (Ser3, Ser223, Thr233, Ser383, and Ser440) to inhibit lipophagy (Nazio et al., 2013; Shimobayashi and Hall, 2014). PI3P generated by the Class III PI3K complex is required for autophagy in yeast and mammals (Burman and Ktistakis, 2010). For example, the ATG18 direct homologs WIPI1 and WIPI2 in mammalian cells can participate in autophagy by binding to PI3P under starvation or amino acid deficiency conditions (Polson et al., 2010). Moreover, WIPI2 has been shown to function by binding directly to ATG16-L1 in the ATG12-ATG5-ATG16-L1 complex associated with the ubiquitin-like protein binding system to form autophagosomes. mTORC1 has been shown to phosphorylate the Ser395 site of WIPI2 and induce specific interaction of WIPI2 with the E3 ubiquitin ligase HUWE1 to control autophagosome formation (Dooley et al., 2015; Wan et al., 2018).

mTORC1 also has significant effects on autophagosome and lipophagy gene related transcription factors. It has been found that under nutrient-rich conditions, mTORC1 phosphorylates ribosomal protein S6 kinase (S6K2), forming a complex with nuclear receptor co-repressor 1 (NCoR1). In addition, mTORC1 binds to and phosphorylates transcription factor EB (TFEB), the master transcription factor responsible for ATG and lysosomal genes, remaining in the cytoplasm. In contrast, in the absence of nutrients or inactivation of mTORC1, nuclear translocation of NCoR1 is inhibited. TFEB is subsequently dephosphorylated and enters the nucleus to participate in the transcription of lysosomal genes. ULK1 kinase is activated, which in turn induces lipophagy. Upon activation of lipophagy, on the one hand, peroxisome proliferator-activated receptor α (PPARα) is activated by degrading NCoR1. On the other hand, activated PPARα upregulates lipophagy through AMPK/mTOR signaling, decreasing intrahepatic lipid content and stimulating *ß*-oxidation in the liver and hepatocytes (Martina et al., 2012; Saito et al., 2019; Tong et al., 2019). Furthermore, studies have shown that PPARα and farnesol-like X receptor (FXR) compete for the same DNA binding site in the ATG gene promoter with opposite transcriptional outputs and that pharmacological activation of PPARα reverses the normal inhibition of lipophagy in the fed state and induces lipid degradation (Lee et al., 2014a).

mTORC1 also has downstream negative regulators of lipophagy. Koren first indicated that death-associated protein 1 (DAP1) is involved in the negative regulation of autophagy. Further studies showed that mTOR is a specific kinase of DAP1 (Koren et al., 2010; Yahiro et al., 2014). In addition, mTORC1 has also been shown to inhibit autophagosome formation through direct phosphorylation and activation of acetyltransferase p300. Acetyltransferase p300 has been identified as a direct phosphorylation substrate for mTORC1. mTOR phosphorylates and activates four serine residues (Ser2271, Ser2279, Ser2291 and Ser2315) in the C-terminal structural domain of p300 (Wan et al., 2017).

mTOR has been found to have a significant role in the terminal phase of lipophagy. mTORC1 phosphorylates the Ser498 site of UVRAG to prevent the maturation of autophagosomes (Kim et al., 2015). And during the sustained starvation response, autophagosomes formed by the fusion of autophagosomes with lysosomes can reactivate mTOR, providing negative feedback to inhibit further autophagosome formation and prevent the breakdown of more components than necessary for survival (Munson and Ganley, 2015).

## Targeting mTORC1-dependent lipophagy for the treatment of NAFLD

### Lipophagy and hepatic steatosis

Triglycerides stored in LDs, which accumulate in large amounts to initiate hepatocyte steatosis, are essential triggers of NAFLD (Canbay et al., 2007; Scorletti and Carr, 2022). Targeting mTORC1-regulated lipophagy to promote LDs degradation may be an effective therapeutic strategy to improve NAFLD (Filali-Mouncef et al., 2022).

In NAFLD, enhanced lipophagy can directly affect *de novo lipogenesis* (DNL). For example, perinatal exposure to bisphenol A (BPA) causes NAFLD in both female and male offspring, which is associated with the inhibition of lipophagy *via* the AKT/mTOR signaling pathway, leading to the upregulation of lipogenic genes (Lin et al., 2019). In addition, extensive studies have found that inhibition of lipophagy exacerbates hepatic lipid accumulation, which is highly correlated with sustained activation of mTORC1 in the liver of high-fat-fed mice (Korsheninnikova et al., 2006). Pharmacological intervention with the autophagy inducers rapamycin or carbamazepine significantly reduced liver, blood triglyceride, glucose, and plasma insulin levels in high-fat diet-fed mice, indicating an improvement in NAFLD by restoring lipophilic flux through inhibition of the mTOR-dependent pathway (Lin et al., 2013). Acyl coenzyme A oxidase 1 (Acox1), an enzyme that catalyzes the first step in peroxisome *ß*-oxidation, accumulates in the liver and further increases during fasting or a high-fat diet (HFD). Liver-specific Acox1 knockout (Acox1-LKO) protects mice from hepatic steatosis induced by starvation or HFD, which is associated with reduced lysosomal localization of mTOR, leading to impaired activation of mTORC1 and induction of lipophagy (He et al., 2020). In HFD-fed mouse models of early hepatic steatosis and L02 cells with A/O-induced lipid hyperaccumulation, Caveolin1 (CAV1) promotes lipophagy by inhibiting the AKT/mTOR pathway, which attenuates lipid accumulation (Xue et al., 2020). Casein kinase 2-interacting protein 1 (CKIP-1), a positive regulator of autophagy in hepatocytes, activates lipophagy by inhibiting AKT/mTOR signaling, thereby reducing lipid accumulation (Li et al., 2020a).

AMP-dependent protein kinase (AMPK) can inhibit mTOR directly or indirectly, but its activity is reduced in NAFLD (Jacquel et al., 2018; Zhao et al., 2020). Restoring lipophagy by increasing AMPK activity is also an important target for improving NAFLD. For example, the glucagon-like peptide 1 (GLP-1) analog liraglutide (LRG) restores lipophagy by increasing AMPK activity, ultimately improving hepatocyte steatosis (He et al., 2016). Chemotherapeutic isothiocyanate radixanthin (SFN) contributes to lipophagy in adipocytes of mice fed HFD and induces lipophagy *via* the AMPK-mTOR-ULK1 signaling pathway in differentiated 3T3-L1 cells (Masuda et al., 2022).

The imbalance between lipogenesis and lipolysis may lead to steatosis, and lipophagy is essential in maintaining lipid homeostasis; focusing on the extended side may be the key to NAFLD treatment. Regulation of lipophagy by targeting the mTOR signaling pathway is critical in adipogenesis and lipid metabolism, and is an essential target for the treatment of NAFLD.

### Lipophagy and insulin resistance

The development of NAFLD is closely related to insulin resistance. On the one hand, hepatic fat accumulation leads to insulin resistance, and on the other hand, insulin resistance increases serum-free fatty acid levels by promoting lipolysis (Zhang et al., 2018; Watt et al., 2019). In addition, fat accumulation decreases lipophagic flux, leading to defective insulin action (Yang et al., 2010).

It was tentatively suggested that enhancing lipophagy by inhibiting mTOR has an ameliorative effect on insulin resistance: the autophagy inducer rapamycin ameliorated reduced glucose tolerance, decreased insulin sensitivity, dysglycemia, and dyslipidemia, increased hepatic steatosis, enhanced hepatic inflammation, elevated p-mTOR expression and decreased lipophagic flux caused by HFD and STZ-induced T2DM rats, and The autophagy inhibitor 3-MA significantly reversed the effects of rapamycin, which suggested that rapamycin improved insulin resistance and hepatic steatosis in T2DM rats by activating lipophagy, but the upstream molecular mechanism of mTOR was not elucidated (Zhou and Ye, 2018). In addition, bee pollen polysaccharides alleviated hepatic steatosis and insulin resistance in HFD-fed mice by promoting lipophagy through an AMPK/mTOR-dependent signaling pathway. Sitagliptin significantly improved hepatic steatosis and insulin resistance in ob/ob mice by activating lipophagy through the AMPK/mTOR signaling pathway (Li et al., 2017; Zheng et al., 2018). More studies in the future are still needed to elucidate the role of lipophagy in hepatic selective insulin resistance, thus providing new directions and perspectives for the prevention and treatment of NAFLD.

### Lipophagy and liver fibrosis

Hepatic fibrosis is a pathological feature of various chronic liver diseases, including NAFLD, characterized by persistent extracellular matrix (ECM) accumulation (Parola and Pinzani, 2019). Substantial evidence indicates that hepatic stellate cells (HSCs) are an essential source of ECM and that their activation is a critical event in the development of liver fibrosis (Elpek, 2014). Therefore, inhibiting the activation of HSCs is considered a vital strategy to reverse liver fibrosis.

Lipophagy has an essential role in liver fibrosis. On the one hand, it has been proposed that lipophagy promotes the activation of hematopoietic stem cells. The main feature of hematopoietic stem cell activation is the release of LDs containing retinol and triglycerides. Meanwhile, lipophagy degrades lipid droplets in hematopoietic stem cells, which hydrolyzes retinol into free fatty acids, further oxidized by mitochondria to generate ATP, providing energy for cell activation (Hernandez-Gea and Friedman, 2012). The insulin-like growth factor binding protein-related protein (IGFBPrP1) has also been shown to upregulate lipophagy by inhibiting the PI3K/AKT/mTOR signaling pathway, thus promoting the activation of hematopoietic stem cells (Huang et al., 2019).

On the other hand, lipophagy has also been suggested to inhibit liver fibrosis. Rutin and curcumin stimulate fatty acid-induced lipophagy in NHSCs (non-chemically induced HSCs) by regulating the PI3K/AKT/mTOR pathway, which ultimately inhibits the activation of NHSCs (Lee et al., 2014b).

The above results suggest the importance of mTOR-regulated lipophagy in liver fibrosis. However, it is unclear why the different effects might be due to varying degrees of lipophagy activation in HSC and thus other effects: moderate activation of lipophagy in HSC cells could provide energy for HSC activation. Conversely, excessive activation of HSC lipophagy might lead to the aging and death of HSC autophagy cells, thus inhibiting HSC activation. Therefore, more studies are still needed to explore the relationship between them.

## Conclusion and perspectives

mTORC1, as a central negative regulator of adipose autophagy, exerts an essential regulatory role on the initiation, formation, and end stages of lipophagy and is a highly effective therapeutic target. Moreover, restoration of lipophagy dysregulation based on mTORC1 plays different effects in different stages of NAFLD development. In the early stage of NAFLD progression, the leading role of lipophagy is to degrade LDs in hepatocytes to alleviate steatosis and thus improve NAFLD. Although lipophagy is thought to provide binding energy for the activation of hepatic fibroblasts in the later stages of NAFLD development, enhanced autophagy has also been shown to inhibit the activation of HSCs and thereby ameliorate liver fibrosis. Therefore, accurately assessing the progress stage of NAFLD and targeting the intervention of lipophagic signaling molecules in hepatocytes are more conducive to the treatment of NAFLD. Lipophagy makes essential contributions in regulating hepatic mass metabolism and maintaining energy homeostasis and also provides valuable novel ideas for the therapeutic approach of NAFLD. Multiple molecular mechanisms regulate lipophagy, and in this paper, we discuss mTORC1-centered lipophagy to provide a candidate strategy for the clinical treatment of NAFLD.
